# Role of Homeobox (HOX) Genes in Adult Cardiovascular Disease: From Developmental Regulators to Therapeutic Targets

**DOI:** 10.7759/cureus.95730

**Published:** 2025-10-30

**Authors:** Akhil Bharadwaz Gangula, Likhitha Kona

**Affiliations:** 1 General Practice, Prathima Institute of Medical Sciences, Hyderabad, IND; 2 Internal Medicine, Seven Hills Hospital, Visakhapatnam, IND

**Keywords:** atherosclerosis, cardiac fibrosis, cardiovascular disease, homeobox genes, precision medicine, transcriptional regulation

## Abstract

Homeobox (HOX) genes, once primarily viewed as regulators of embryonic development, are now acknowledged as important factors in adult cardiovascular pathophysiology. This narrative review consolidates existing knowledge from developmental biology, molecular cardiology, and translational medicine to clarify the roles of HOX genes in cardiovascular disease, emphasizing molecular mechanisms and therapeutic ramifications. HOX genes operate as context-dependent molecular switches, exhibiting unique roles specific to various diseases. HOXA5 safeguards against atherosclerosis via peroxisome proliferator-activated receptor gamma (PPARγ), whereas HOXB9 exacerbates inflammation through bone morphogenetic protein 4-tumor necrosis factor (BMP4-TNF). HOXA4 demonstrates dual functionality, safeguarding against vascular remodeling while facilitating cardiac fibrosis. Functional specificity is attained via trimeric complexes comprising pre-B-cell leukemia homeobox (PBX) and myeloid ecotropic viral integration site (MEIS) proteins, with epigenetic modifications facilitating dynamic regulation. Therapeutic strategies, such as HOX-PBX inhibition, epigenetic modulation, and gene therapy, exhibit encouraging preclinical outcomes. HOX genes serve as principal regulators of the cardiovascular system, and their dysfunction markedly influences disease development, thereby establishing HOX-targeted therapies as innovative precision medicine strategies necessitating expedited clinical translation.

## Introduction and background

Cardiovascular disease continues to be the primary cause of global mortality, influenced by intricate genetic, epigenetic, and environmental factors that require novel therapeutic strategies aimed at fundamental pathogenic mechanisms rather than merely addressing downstream effects [1]. Recent advancements in multi-omics profiling have facilitated the identification of critical gene regulatory networks that underpin cardiovascular pathophysiology, including the sustained activity of developmental transcription factors in adult disease conditions [2].

Homeobox (HOX) genes encode a conserved family of transcription factors characterized by a 60-amino-acid homeodomain that mediates sequence-specific DNA binding. The human genome contains 39 HOX genes, organized into four clusters: HOXA, HOXB, HOXC, and HOXD. These genes are highly conserved across evolutionary history [3]. Although initially studied in the context of embryonic development, recent evidence suggests that they play persistent regulatory roles in adult cardiovascular biology. Dysregulation of HOX genes contributes to the development of atherosclerosis, cardiac fibrosis, vascular remodeling, and ischemic heart disease [4,5].

Rationale for narrative review approach

The complex nature of HOX gene biology within cardiovascular contexts necessitates a narrative review approach that is adept at addressing the inherent disciplinary diversity and interpretive challenges. Given that HOX research spans developmental biology, molecular cardiology, vascular biology, and translational medicine, each employing distinct methodological frameworks that preclude quantitative meta-analysis, this narrative method is uniquely suited to synthesizing disparate evidence. This approach aims to elucidate critical questions: how HOX genes transition from developmental regulators to contributors to disease, what factors dictate their context-dependent protective or detrimental roles, and how therapeutic strategies targeting these genes can be optimized for clinical efficacy.

Scope and objectives

This review synthesizes current knowledge concerning the role of HOX genes as critical regulators in the advancement of cardiovascular diseases, extending beyond simple gene identification to encompass intricate regulatory networks. The focus is placed on HOX gene functions in the context of adult cardiovascular pathophysiology, demonstrating that the transcriptional regulatory networks established during development persist and influence disease susceptibility and progression throughout an individual's lifespan.

## Review

Methodology

Literature Selection Strategy and Inclusion Criteria

A purposive sampling strategy, typical of narrative reviews, was utilized to identify studies elucidating the roles of HOX genes in cardiovascular contexts. This approach was chosen due to the interdisciplinary scope of HOX gene research, which spans developmental biology, molecular cardiology, and translational medicine, and often precludes systematic quantitative synthesis. The literature search included studies identified using keywords such as 'homeobox,' 'HOX genes,' 'cardiovascular disease,' 'atherosclerosis,' 'cardiac fibrosis,' and 'vascular remodeling' across PubMed/Medical Literature Analysis and Retrieval System Online (MEDLINE), Excerpta Medica database (Embase), and Web of Science databases, covering publications from 2018 through June 2025. Studies were prioritized if they addressed HOX protein mechanisms, translational or clinical evidence of disease association, therapeutic targeting strategies, or novel conceptual frameworks. Studies focusing exclusively on embryonic development without relevance to adult disease were excluded.

Molecular mechanisms governing HOX gene function

Transcriptional Regulation and DNA Binding Specificity

HOX proteins are transcription factors that bind to specific DNA sequences. They have a 60-amino-acid homeodomain that recognizes the TAAT core DNA sequence [6]. Heterodimerization with pre-B-cell leukemia homeobox (PBX) cofactors, which are members of the three amino acid loop extension (TALE) family, dramatically improves the specificity of HOX protein-DNA interactions. This is because PBX cofactors can recognize extended and bipartite DNA motifs, which makes target selectivity and functional diversity higher [7].

The functional specificity of HOX proteins in cardiovascular biology is dictated by their interactions with TALE family cofactors, particularly PBX and myeloid ecotropic viral integration site (MEIS) proteins. The HOX hexapeptide motif facilitates cooperative binding between HOX and PBX by fitting into a hydrophobic pocket created by PBX1 homeodomain residues, thereby increasing both DNA binding affinity and specificity [8]. This mechanism allows HOX-PBX complexes to differentiate closely related target sequences, with paralog-specific residues further influencing interactions.

MEIS proteins are essential for conferring a third dimension to HOX regulatory complexes by forming trimeric assemblies with distinct functional properties. MEIS1 modulates HOX-PBX interactions, often rendering the hexapeptide motif dispensable for complex formation and enhancing DNA binding efficiency [9]. MEIS proteins regulate chromatin accessibility; MEIS1 recruits CREB-binding protein (CBP) acetyltransferase to acetylate histones and activate gene expression, whereas PBX recruits histone deacetylases to repress transcription [10]. This competitive interaction constitutes a central mechanism by which HOX-regulated promoters alternate between transcriptional activation and repression.

Epigenetic Control Mechanisms

Epigenetic mechanisms, especially DNA methylation, tightly control HOX gene expression in cardiovascular tissues. These mechanisms combine developmental programming with environmental factors and disease states. DNA methylation at HOX gene clusters functions as a long-term memory system, maintaining transcriptional states formed during development and facilitating pathological reactivation in adult cardiovascular disease scenarios [11].

Clinical studies have shown that changes in the methylation patterns of HOX genes are associated with heart disease. In thoracic aortic dissection, HOXA5 hypermethylation is associated with lower gene expression and compromised aortic wall integrity, functioning as a clinical-grade biomarker for disease detection with 86% sensitivity and 75% specificity [12]. These findings support the concept that epigenetic modifications of HOX genes function both as disease drivers and diagnostic biomarkers.

Disease-specific roles: resolving functional contradictions

Atherosclerosis: Context-Dependent Protective and Pathogenic Functions

HOX genes play distinct roles in atherosclerosis, with varying members exhibiting either protective or detrimental effects, depending on the type of cell and the stage of the disease. HOXA5 protects cells by maintaining vascular smooth muscle cells (VSMCs) in a contractile state and activating peroxisome proliferator-activated receptor gamma (PPARγ) signaling. HOXA5 overexpression inhibits VSMC dedifferentiation and migration, diminishes neointimal hyperplasia, and mitigates plaque formation. In contrast, pharmacological PPARγ inhibition reverses these effects, thereby substantiating the mechanistic connection [13]. HOXA5 also promotes M2 macrophage polarization through the transcriptional activation of MED1, thereby contributing to its anti-atherogenic effects [14].

On the other hand, HOXB9 causes atherosclerotic inflammation by activating the bone morphogenetic protein 4 (BMP4) and tumor necrosis factor (TNF) signaling pathways. HOXB9 is upregulated in endothelial cells subjected to low wall shear stress. It facilitates the induction of inflammatory mediators through BMP4-dependent TNF and nuclear factor kappa B (NF-κB) activation, especially at atheroprone locations [15].

The temporal dynamics of HOX gene expression demonstrate distinct patterns in early and advanced atherosclerotic lesions. In advanced lesions, HOXA5 expression is lower, which is associated with increased VSMC plasticity and inflammation. On the other hand, HOXB9 and other pro-inflammatory HOX genes are more active in areas with disturbed flow [13,15]. These results suggest that therapeutic approaches should be tailored to the stage of the disease: augmenting HOXA5 activity may be beneficial in early disease to maintain vascular stability, whereas suppressing HOXB9 may prove more efficacious in advanced lesions.

The HOXA4 Paradox: Tissue-Specific Function Resolution

Recent advancements have elucidated the dual and context-dependent roles of HOXA4 in cardiovascular remodeling, exhibiting specific functions in vascular smooth muscle cell biology and cardiac fibrosis. In vascular tissues, HOXA4 inhibits maladaptive remodeling by directly repressing the transcriptional activity of yes-associated protein/transcriptional enhancer activator domain (YAP/TEAD). HOXA4 interacts with TEAD transcription factors, competing with YAP for TEAD binding and thereby reducing gene expression that is mediated by YAP and TEAD. This interaction maintains VSMC differentiation and contractile phenotype stability, preventing phenotypic switching and excessive vascular remodeling [16].

In contrast, HOXA4 acts as a profibrotic factor in the context of cardiac fibrosis. The MIAT/miR-150/HOXA4 axis has been recognized as a principal regulatory mechanism in ischemic heart failure. Myocardial infarction-associated transcript (MIAT), a long non-coding RNA upregulated in failing hearts, inhibits microRNA-150 (miR-150), which typically suppresses HOXA4 expression. Overexpressing HOXA4 exacerbates remodeling after a heart attack, whereas overexpressing miR-150 protects against fibrosis [17]. This context-dependent characteristic, protective in vascular remodeling and profibrotic in cardiac injury, illustrates the intricacy of HOX gene regulation and the impact of the cellular microenvironment.

Cardiac Fibrosis and Ischemic Heart Disease

In cardiac pathophysiology, HOX genes exhibit specific, context-dependent functions that correspond to the heart's unique developmental and functional needs. HOXA4 serves as a principal pathogenic factor in cardiac fibrosis, facilitating both fibroblast proliferation and activation after myocardial injury. Mechanistic studies demonstrate that HOXA4 is upregulated in cardiac fibroblasts following ischemic injury, with HOXA4 deficiency protecting against maladaptive remodeling. Clinical studies have validated the upregulation of HOXA4 in human heart failure, with expression levels correlating with disease severity [17].

HOXA3 provides contrasting protective functions in the injured heart. HOXA3 is upregulated by growth differentiation factor 11 (GDF11) and mediates inhibition of cardiomyocyte pyroptosis following myocardial infarction. HOXA3 directly regulates components of the nucleotide-binding oligomerization domain-like receptor pyrin domain-containing 3 (NLRP3) inflammasome, thereby modulating inflammatory responses and limiting cell death in the post-infarct myocardium [18]. The different and opposite effects of HOXA4 and HOXA3 demonstrate the complexity of HOX gene function and their potential applications in treating fibrosis, inflammation, and adverse remodeling.

Therapeutic targeting strategies

HOX-PBX Inhibition Approaches

HOX-PBX protein interactions are essential to the pathological gene expression programs in cardiovascular disease. The formation of HOX-PBX heterodimers significantly enhances DNA-binding specificity and affinity, allowing for precise control of gene networks involved in atherosclerosis and cardiac fibrosis [19]. Disruption of these complexes, particularly at the hexapeptide recognition site on PBX proteins, has emerged as a promising therapeutic approach.

Peptide inhibitors, such as HTL001 and HXR9, bind to the PBX hexapeptide pocket in a competitive manner. This stops HOX-PBX dimerization and changes the transcriptional programs that follow. Preclinical studies demonstrate that these peptides induce apoptosis and alter the disease course in models where HOX-PBX signaling is impaired [20]. Peptide-based platforms are highly specific, allowing them to target diseased tissues but not healthy ones. In cardiovascular settings, HOX-PBX inhibition has been shown to regulate fibroblast activation, inflammatory signaling, and vascular remodeling [21].

Epigenetic Modulation Strategies

Epigenetic methods, such as DNA methyltransferase inhibitors like azacytidine and decitabine, have been shown in clinical studies to reactivate HOX gene expression. While the majority of clinical data originates from cancer trials, these agents demonstrate that the demethylation of HOX genes post-treatment correlates with enhanced clinical outcomes, thereby providing proof of concept for HOX-targeted epigenetic therapy suitable for cardiovascular applications [22].

Histone deacetylase inhibitors, such as valproic acid, provide an additional pathway for reactivating HOX genes through chromatin remodeling. Valproic acid exhibits advantageous effects in preclinical models of heart failure and myocardial infarction, encompassing anti-inflammatory, anti-fibrotic, and cardioprotective properties, with specific mechanisms involving the modulation of HOX gene expression [23]. The reversibility and specificity of these epigenetic drugs render them promising candidates for repurposing in cardiovascular conditions where HOX gene reactivation is therapeutically advantageous.

Gene Therapy Innovations

Antisense oligonucleotide (ASO) platforms are a rapidly growing method for modulating the activity of specific genes. Chemical modifications, such as phosphorothioate backbones and 2'-O-methoxyethyl ribose substitutions, are common in today's ASO drugs. These modifications enhance nuclease resistance, improve tissue penetration, and reduce immunogenicity [24]. Delivery methods, such as direct cardiac injection, systemic administration, and nanoparticle-based formulations, enable more targeted delivery to tissues and allow for controlled release in cardiovascular applications [25].

Clustered regularly interspaced short palindromic repeats (CRISPR)-based genome editing, encompassing base editing technologies, facilitates accurate single-nucleotide alterations devoid of double-strand breaks. VERVE-101, a CRISPR-based editor targeting proprotein convertase subtilisin/kexin type 9 (PCSK9), demonstrates that this approach is feasible. It has been shown to lower low-density lipoprotein (LDL) cholesterol levels in nonhuman primates for a long time and is now being tested in humans for cardiovascular purposes [26]. These developments support the promise of ASO and CRISPR-based strategies for precise, durable, and tissue-specific modulation of HOX genes.

Clinical Biomarker Development

HOX gene expression and methylation patterns are increasingly acknowledged as reliable biomarkers for the diagnosis, prognosis, and therapeutic monitoring of cardiovascular diseases. The most clinically advanced application utilizes cell-free DNA methylation signatures of HOX genes for the detection of thoracic aortic dissection, wherein the analysis of circulating methylation patterns demonstrates enhanced sensitivity and specificity relative to conventional protein-based biomarkers [12]. In particular, the methylation profiles of HOXA5, HOXB6, and HOXC6 yield tissue-specific signatures that facilitate the differentiation of thoracic aortic dissection from healthy controls.

The stability of DNA methylation marks in circulation and their resilience to acute inflammatory changes establish HOX epigenetic biomarkers as essential tools for evaluating both acute and chronic diseases [27]. Point-of-care testing platforms are being developed for the rapid measurement of HOX methylation biomarkers. These platforms utilize novel molecular diagnostics to facilitate risk stratification and emergency diagnosis.

Table 1 summarizes the key HOX genes implicated in cardiovascular pathophysiology, organizing their context-dependent roles, underlying molecular mechanisms, and therapeutic targeting potential. This synthesis reveals distinct functional patterns that inform rational approaches to HOX-targeted cardiovascular therapeutics.

**Table 1:** HOX Genes in Cardiovascular Disease: Functional Roles, Mechanisms, and Therapeutic Approach Table data compiled from references [12-18,20-26]; HOXA5 data from [12-14,22,23]; HOXB9 data from [15,20,21,24,25]; HOXA4 data from [16,17,25,26]; HOXA3 data from [18,26]. Therapeutic development status based on the current literature review. ASO: antisense oligonucleotide; BMP4: bone morphogenetic protein 4; GDF11: growth differentiation factor 11; HOX: homeobox; MIAT: myocardial infarction-associated transcript; NF-κB: nuclear factor kappa B; NLRP3: nucleotide-binding oligomerization domain-like receptor pyrin domain-containing 3; PPARγ: peroxisome proliferator-activated receptor gamma; TEAD: transcriptional enhancer activator domain; TNF: tumor necrosis factor; VSMC: vascular smooth muscle cell; YAP: yes-associated protein

HOX Gene	Cardiovascular Role	Molecular Mechanism	Disease Context	Therapeutic Approach	Development Status
HOXA5	Protective; Anti-atherosclerotic; Vascular homeostasis	PPARγ pathway activation; VSMC contractile phenotype maintenance; M2 macrophage polarization via MED1; Anti-inflammatory signaling	Atherosclerosis prevention; Neointimal hyperplasia; Vascular remodeling; Thoracic aortic dissection	Activation strategies: PPARγ agonists, demethylating agents; Biomarker applications: Methylation-based diagnostics; Gene therapy: Overexpression vectors	Clinical biomarkers (86% sensitivity, 75% specificity); Therapeutic activation (Preclinical)
HOXB9	Pathogenic; Pro-atherogenic; Endothelial dysfunction	BMP4-TNF signaling activation; NF-κB pathway stimulation; Inflammatory mediator induction; Low wall shear stress response	Atherosclerosis progression; Endothelial inflammation; Atheroprone vascular sites; Advanced lesion formation	HOX-PBX inhibition: HTL001, HXR9 peptides; Epigenetic silencing: DNA methylation targeting; Antisense therapy: ASO-mediated knockdown	Peptide inhibitors (Preclinical models); ASO platforms (Development phase)
HOXA4	Dual function; Context-dependent; Tissue-specific roles	Vascular: YAP/TEAD inhibition, VSMC stabilization; Cardiac: MIAT/miR-150 axis, fibroblast activation; Competitive TEAD binding	Protective: Vascular remodeling prevention; Pathogenic: Cardiac fibrosis, heart failure; Ischemic heart disease	Tissue-specific targeting: Cardiac-selective inhibition; miRNA therapy: miR-150 restoration; Context-aware modulation: Disease stage-specific approaches	Tissue-selective targeting (Research phase); miRNA therapeutics (Early development)
HOXA3	Protective; Cardioprotective; Anti-pyroptotic	NLRP3 inflammasome regulation; Cardiomyocyte pyroptosis inhibition; GDF11-mediated upregulation; Inflammatory response modulation	Myocardial infarction; Ischemia-reperfusion injury; Post-infarct remodeling; Heart failure prevention	GDF11 pathway targeting: Growth factor therapy; Direct activation: HOXA3 overexpression; Pyroptosis inhibition: Inflammasome modulators	GDF11 therapies (Preclinical validation); Gene therapy vectors (Research phase)

Future perspectives and clinical translation

Integration with Precision Medicine

The combination of cutting-edge genomic technologies and a better understanding of HOX biology opens up new possibilities for precision cardiovascular medicine. Patient stratification based on HOX gene profiles signifies a transformative shift towards personalized care, facilitating targeted interventions for individuals exhibiting distinct HOX dysregulation patterns [28].

Single-cell RNA sequencing (scRNA-seq) technologies are transforming the comprehension of HOX gene function by uncovering cell-type-specific expression patterns and regulatory networks that were previously obscured by bulk tissue analysis [29]. These methods enable the identification of rare cell populations, the mapping of cellular heterogeneity, and the determination of lineage trajectories that are important for heart development and disease.

Integrating single-cell data with spatial transcriptomics enables high-resolution mapping of HOX gene expression in intact cardiovascular tissues, offering critical insights into the influence of cellular organization and tissue architecture on gene function [30]. These technologies drive innovation in computational analysis, deep learning, and multi-omics integration, thereby advancing precision medicine and enhancing the clinical relevance of HOX biology.

Computational Approaches and Systems Biology

Machine learning methods are becoming more helpful in finding HOX gene signatures that can predict heart problems. Combining HOX expression data with electronic health records and multi-omics profiles enables the creation of real-world evidence and the identification of previously unknown links [31]. These computational approaches facilitate the discovery of novel therapeutic targets and the prediction of individual patient responses to HOX-targeted interventions.

Network-based analyses elucidate the role of HOX genes within extensive regulatory networks that control cardiovascular biology, offering insights into the molecular mechanisms that drive disease heterogeneity and progression [32]. Comprehending these network characteristics facilitates the identification of combinatorial therapeutic strategies that simultaneously target multiple nodes, potentially yielding superior efficacy compared to single-target methodologies.

Limitations and Future Research Directions

Current limitations in HOX gene cardiovascular research encompass an inadequate mechanistic comprehension of regulatory networks, context-dependent gene functionalities, and difficulties in translating molecular discoveries into clinical applications [4]. The variability of HOX gene expression among cardiovascular cell types and disease conditions hinders biomarker development and therapeutic targeting. Furthermore, the functional roles of antisense long non-coding RNAs (lncRNAs) embedded within HOX clusters and their interactions with canonical HOX genes are inadequately characterized [33].

Future research priorities should focus on elucidating the molecular mechanisms underlying HOX gene and HOX-long non-coding RNA (lncRNA) interactions across different cardiovascular contexts, from development through disease progression and regeneration. Advanced genomic technologies, particularly single-cell and spatial transcriptomics, must be leveraged to decode cell-type-specific HOX expression patterns and regulatory networks that drive cardiovascular pathophysiology.

The development and validation of HOX-based biomarkers represents another critical research direction, emphasizing diagnostic, prognostic, and therapeutic monitoring applications with a focus on clinical translation and point-of-care platforms. Simultaneously, the integration of multi-omics data with machine learning methodologies should be advanced to enable personalized risk prediction and targeted therapeutic interventions. These efforts must be complemented by a comprehensive examination of the therapeutic potential of HOX gene modulation through gene editing, antisense oligonucleotides, and small molecule inhibitors in both preclinical and clinical models. Addressing these limitations will be essential for realizing the full potential of HOX gene biology in precision cardiovascular medicine.

## Conclusions

HOX genes are fundamental regulators within cardiovascular biology, governing complex pathophysiological processes from embryonic development to adult cardiovascular diseases. This review posits HOX genes as sophisticated, context-dependent molecular switches that integrate various cellular signals to elicit specific biological responses, thereby holding significant potential for advancing precision cardiovascular medicine. Their multifaceted roles present intricate therapeutic landscapes: HOXA5 demonstrates protective attributes amenable to therapeutic enhancement, whereas HOXB9 and dysregulated HOXA4 contribute to pathological states, making them viable targets for intervention. Further mechanistic studies are essential to resolve functional discrepancies and inform rational therapeutic design that respects the complex characteristics of HOX regulatory networks.

In the coming decade, current therapeutic development pathways are expected to yield promising clinical applications. Epigenetic modulation techniques are poised for near-term implementation, while antisense oligonucleotide platforms offer prospects for refined therapeutic precision in the intermediate term. The combination of advanced genomic technologies and robust regulatory frameworks is driving significant advancements in precision cardiovascular medicine. HOX-targeted therapies are anticipated to become critical interventions, significantly improving outcomes for individuals worldwide affected by cardiovascular conditions. Elucidating and harnessing intricate HOX regulatory networks holds the potential to revolutionize heart disease prevention, diagnosis, and treatment. Achieving these advancements requires ongoing interdisciplinary collaboration, bridging basic developmental biology and clinical investigation. This will translate fundamental discoveries into improved patient care through comprehensive, integrated strategies that fully utilize the therapeutic potential of these master cardiovascular regulators.
